# Cell-Free and Concentrated Ascites Reinfusion Therapy during Hemodialysis for Intradialytic Hypotension and Intractable Ascites

**DOI:** 10.1155/2022/7099227

**Published:** 2022-10-15

**Authors:** Hideyuki Hayasaka, Kiyonori Ito, Susumu Ookawara, Masaya Kofuji, Takayuki Uchida, Shunto Kawamura, Ayumi Gomyo, Haruhisa Miyazawa, Yuichiro Ueda, Keiji Hirai, Shun-ichi Kimura, Naoki Momose, Shinichi Kako, Yoshiyuki Morishita

**Affiliations:** ^1^Department of Clinical Engineering, Saitama Medical Center, Jichi Medical University, Saitama, Japan; ^2^Division of Nephrology, First Department of Integrated Medicine, Saitama Medical Center, Jichi Medical University, Saitama, Japan; ^3^Division of Hematology, First Department of Integrated Medicine, Saitama Medical Center, Jichi Medical University, Saitama, Japan

## Abstract

A 60-year-old woman with POEMS (polyneuropathy, organomegaly, endocrinopathy, monoclonal gammopathy, and skin changes) syndrome and intractable ascites presented with acute renal failure and received hemodialysis (HD) therapy. Due to frequent intradialytic hypotension, ultrafiltration with cell-free and concentrated ascites reinfusion therapy (CART) was performed to adequately manage the body fluid status and massive ascites. During HD with CART, her blood pressure was maintained compared with that during HD without CART, and an ultrafiltration volume of 3.7 L was achieved after HD with CART. In HD patients with intradialytic hypotension and massive ascites, the combination of CART and ultrafiltration during HD may be an effective therapeutic option for body-fluid management.

## 1. Introduction

Intradialytic hypotension (IDH) is a fatal and dangerous complication in hemodialysis (HD) patients [1]. Therapeutic strategies against IDH include setting an appropriate dry weight, evaluating cardiac function, and increasing serum protein by improving the nutritional status [2]. In addition, vasopressors or albumin may be administered to perform ultrafiltration during HD therapy.

Cell-free and concentrated ascites reinfusion therapy (CART) is employed for patients with massive ascites and has been implemented for more than 30 years in Japan [3]. CART is divided into three steps. First, there is aspiration of the massive ascites from the patient's abdominal cavity. Next, cells, such as red blood cells or white blood cells, are removed from the collected ascites, and ascites is concentrated by removing the excess fluid. Finally, cell-free and concentrated ascites is reinfused intravenously. The therapeutic effects of CART include amelioration of diuretic resistance, improvement in abdominal tension and quality of life, as well as an adjustment of body fluid status [4, 5]. Furthermore, CART may lead to a reduction in albumin administration, which is more economical (medically) [6].

Polyneuropathy, organomegaly, endocrinopathy, monoclonal gammopathy, and skin change (POEMS) syndrome is a rare refractory disease. POEMS is associated with various symptoms, including massive ascites caused by the overproduction of the vascular endothelial growth factor (VEGF) [7] and has also been reported to lead to the deterioration of renal function and the development of acute renal failure [8]. In our case, HD was performed for the management of acute renal failure and body fluid excess, including massive ascites. However, IDH frequently occurred during HD with ultrafiltration. Therefore, to prevent the IDH occurrence in response to ultrafiltration, ultrafiltration with CART during HD was performed. Herein, we report a case of successfully managing body fluid excess and massive ascites without the development of IDH by combining CART with ultrafiltration during HD.

## 2. Case Description

**Figure 1:**
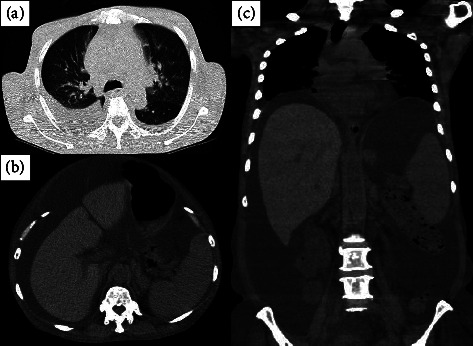
Computed tomography images, (a) lung image in transverse plane, (b) abdominal image in the transverse plane, and (c) thoracoabdominal image in the coronal plane.

**Figure 2:**
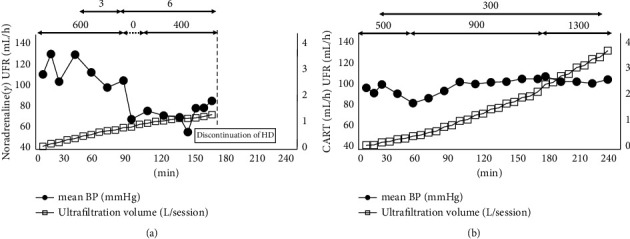
Differences in mean blood pressure and ultrafiltration volume during hemodialysis with or without CART. Abbreviations: BP, blood pressure; CART, cell-free and concentrated ascites reinfusion therapy; HD, hemodialysis; UFR, ultrafiltration rate. (a) HD without CART and (b) HD with CART.

A 60-year-old woman presented with repeated postmenopausal vaginal bleeding due to uterine myoma 3 months prior to admission. Her abdominal computed tomography (CT) was performed at her local hospital to evaluate the uterine myoma and coincidentally showed massive ascites with cirrhotic liver changes. Diuretics were administered in the management of her massive ascites. Thereafter, her renal function rapidly deteriorated, and she was admitted to our hospital. On admission, her height and body weight were 151 cm and 48.0 kg, respectively. Her vital signs were as follows: blood pressure (BP), 95/50 mm·Hg; pulse rate, 76 beats/minute; arterial oxygen saturation on pulse oximetry, 99%. Her physical examination revealed abdominal distension without jaundice or leg edema. Her laboratory findings are shown in Table 1, and the main data are as follows: white blood cell 4.56 × 10^3^/*μ*L, hemoglobin 12.3 g/dL, platelet 14.7 × 10^4^/*μ*L, total protein 6.4 g/dL, serum albumin 3.4 g/dL, serum potassium 7.7 mEq/L, blood urea nitrogen (BUN) 191 mg/dL, serum creatinine (Cr) 6.2 mg/dL, uric acid 18.2 mg/dL, C-reactive protein 4.7 mg/dL, and brain natriuretic peptide 61.6 pg/mL (Table 1). Chest *X*-ray on admission showed cardiac enlargement (cardiothoracic ratio, 57%), whilst computed tomography (CT) showed pleural effusion (Figure 1(a)), massive ascites (Figures 1(b), 1(c)), and multiple uterine myomas. After admission, HD was initiated for the treatment of acute renal failure with mineral and electrolyte disorders and was temporarily discontinued because of improvement in renal function. On day 31 of her hospitalization, her repeated laboratory findings confirmed the presence of serum VEGF >2000 pg/mL, IgG-lambda and kappa type M proteins in the immunoelectrophoretic study, and demyelinating polyneuropathy in the peripheral nerves, and she was diagnosed with POEMS syndrome. Although lenalidomide and dexamethasone therapy was initiated for the treatment of POEMS syndrome, her renal function gradually deteriorated (BUN 92 mg/dL, Cr 5.4 mg/dL) with massive ascites. HD was initiated again on day 63 of hospitalization. The details of her HD therapy were as follows: 3 h per session, 3 times per week; blood flow, 150 mL/min; dialysate flow, 500 mL/min; dialyzer, NV-13S® (Toray Medical, Japan); anticoagulant, nafamostat mesylate 30 mg/h. In addition to the progression of renal dysfunction, there was worsening of her body fluid excess, as she now had systemic edema and massive ascites. Ultrafiltration during HD was performed. However, body fluid excess could not be adequately removed because of the frequent IDH occurrence even with the use of vasopressors during HD on hospital day 80 (Figure 2(a)). To reduce the body fluid excess and prevent IDH occurrence, ultrafiltration with CART during HD was performed on hospital day 82 (Figure 2(b)). The procedure for ascites filtration and concentration was initiated using massive ascites (6.52 L) collected by intraperitoneal puncture. Aspiration of the massive ascites was performed slowly for more than 4 h to prevent the occurrence of hypotension; her systolic BP was maintained within 115 to 140 mm·Hg. Furthermore, the collected ascites was exudative ascites (serum albumin, 2.4 g/dL; ascites albumin, 1.7 g/dL; serum-ascites albumin gradient, 0.7 g/dL). The volume of the filtered concentrated ascites was 884 mL, which contained ascites albumin of 9.6 g/dL and ascites total protein of 16.2 g/dL. The concentrated ascites was stored in the refrigerator overnight and reinfused intravenously during the next HD therapy. The details of CART were as follows: filtration rate, 50 mL/min; processing time, 180 min/session; device name, ACH-Σ® (Asahi Kasei Medical Co., Japan); filter, AHF-MOW (Asahi Kasei Medical Co., Japan); concentrator, AHF-UP (Asahi Kasei Medical Co., Japan). During HD with CART, BP was maintained even under the faster ultrafiltration rate in comparison to what it is in HD without CART (Figure 2(b)). In this HD session with CART, the administrated albumin was consistent with around 85 g; therefore, her serum albumin concentration increased from 2.4 g/dL to 4.0 g/dL due to the albumin administration. In addition, the ultrafiltration volume reached 3.68 L. By removing massive ascites (6.52 L) and performing ultrafiltration (3.68 L) in this HD session with CART, more than 10 L of excessive body fluid was removed. Chemotherapy was continued for the treatment of POEMS syndrome, and her HD therapy was discontinued on hospital day 97 due to the improvement in renal dysfunction. Thereafter, autologous hematopoietic stem cell transplantation was performed, and the patient recovered fully after the bone marrow engraftment.

## 3. Discussion

In HD patients with massive ascites, IDH occurrence is associated with several factors, including a reduction in colloid osmotic pressure (COP), mismatch between ultrafiltration and plasma refilling in response to ultrafiltration during HD, and an increase in intraperitoneal pressure. COP reduction, which is mainly induced by hypoalbuminemia, leads to a decrease in body fluid movement from the interstitium into the intravascular space and was reportedly associated with the occurrence of IDH [1, 9]. Furthermore, the Kidney Disease Outcomes Quality Initiative guidelines recommend an ultrafiltration rate of less than 15 mL/kg/h to prevent IDH [10]; however, our patient developed IDH at an ultrafiltration rate of 8 mL/kg/h even with vasopressor use and excessive body fluid status in HD without CART. In addition, the increase in intraperitoneal pressure induced by massive ascites would be associated with a decrease in venous return to the central circulation, which leads to hemodynamic instability, including a reduction in cardiac output and a decrease in BP. Therefore, in HD patients with massive ascites, the increase in COP during HD and the decrease in intraperitoneal pressure would play an important role in preventing IDH. This would be due to the improvement in plasma refilling in response to ultrafiltration and the increase in venous return to the central circulation.

In this case, the patient's BP increased immediately and was maintained by combining CART and ultrafiltration during HD. Furthermore, the ultrafiltration rate gradually increased during reinfusion of the concentrated ascites, and the ultrafiltration volume reached approximately 3.7 L, which was approximately three times higher than that in HD without CART. Therefore, CART might cause an increase in plasma refilling from the interstitium into the blood vessels by increasing serum albumin concentration [11]. In this case, serum albumin concentration increased from 2.4 to 4.0 g/dL throughout HD with CART. This increase would be in favor of the increase in plasma refilling during HD. Instead of HD therapy with CART, combination therapy with simple ascites removal and albumin preparation might also be a therapeutic option. However, HD therapy with CART could be a better option when the collected ascites is exudative, rather than transudative. This is because the excess proteins, including albumin, could be reinfused in patients with exudative ascites because of the increase in protein concentration in ascites compared to the amount of ready-made albumin preparation. In recent reports, plasma proteins, including serum albumin, have been shown to form a crucial part of the endothelial surface layer [12, 13], which might prevent fluid leakage from blood vessels. It has also been suggested that the anti-inflammatory effects of albumin may contribute to the improvement of vascular permeability [14]. Therefore, concentrated albumin administration with CART might contribute to the promotion of water mobilization into the vascular space and prevention of water extravasation.

Fever, an increase in BP, chest or abdominal pain, and dyspnea are known adverse effects of CART [15]. Particularly, in anuric HD patients, attention should be paid to the increase in BP, congestive heart failure, and pulmonary edema due to the increase in circulating blood volume induced by the increase in plasma refilling with CART, although this patient did not present with these symptoms. In addition, it is important to carefully preserve the filtered concentrated ascites derived from massive ascites. Previous reports have mentioned the possibility of refrigeration or frozen storage of the filtered concentrated ascites [16, 17]. Okamoto et al. reported that there was no change in endotoxin concentrations or properties even after storing ascites at 4°C for 24 h before treatment [16]. If filtered concentrated ascites is stored appropriately, its reinfusion might be considered permissible within 24 h.

In conclusion, we report a case of POEMS syndrome and acute renal failure in a patient who developed IDH and massive ascites. The combination of CART and ultrafiltration during HD could be an effective therapeutic option for body-fluid management in HD patients with massive ascites.

## Figures and Tables

**Table 1:** Laboratory findings on admission.

Laboratory findings	
Total protein, g/dL	6.4
Albumin, g/dL	3.4
Total bilirubin, mg/dL	0.21
Aspartate transaminase, U/L	7
Alanine transaminase, U/L,	4
Lactate dehydrogenase, U/L	149
Alkaline phosphatase, U/L	260
Serum sodium, mEq/L	137
Serum potassium, mEq/L	7.7
Serum chloride, mEq/L	106
Serum corrected calcium, mg/dL	6.2
Serum phosphate, mg/dL	13.2
Serum magnesium, mg/dL	3.3
Blood urea nitrogen, mg/dL	191
Creatinine, mg/dL	6.2
Uric acid, mg/dL	18.2
White blood cells,/*μ*L	4560
Neutrophil, %	85
Lymphocyte, %	5
Monocyte, %	9
Red blood cells, ×10^4^/*μ*L	434
Hemoglobin, g/dL	12.3
Hematocrit, %	37.5
Platelet, ×10^4^/*μ*L	14.7
C-reactive protein, mg/dL	4.7
Blood glucose, mg/dL,	75
Brain natriuretic peptide, pg/mL	61.6

## Data Availability

The data used to support the findings of this study are included within the article.
